# Whole Genome Sequence Analysis of Mutations Accumulated in *rad27***Δ** Yeast Strains with Defects in the Processing of Okazaki Fragments Indicates Template-Switching Events

**DOI:** 10.1534/g3.117.300262

**Published:** 2017-10-03

**Authors:** Sumita Omer, Bar Lavi, Piotr A. Mieczkowski, Shay Covo, Einat Hazkani-Covo

**Affiliations:** * Department of Natural and Life Sciences, The Open University of Israel, 4353701 Ra’anana, Israel; † Department of Plant Pathology and Microbiology, Robert H. Smith Faculty of Agriculture, Food and Environment, Hebrew University, 76100 Rehovot, Israel; ‡ Department of Cell Research and Immunology, George S. Wise Faculty of Life Sciences, Tel Aviv University, 69978, Israel; § Department of Genetics, Lineberger Comprehensive Cancer Center, University of North Carolina at Chapel Hill, North Carolina 27599

**Keywords:** template switching, RAD27, Okazaki fragments, inverted repeats, FEN1

## Abstract

Okazaki fragments that are formed during lagging strand DNA synthesis include an initiating primer consisting of both RNA and DNA. The RNA fragment must be removed before the fragments are joined. In *Saccharomyces cerevisiae*, a key player in this process is the structure-specific flap endonuclease, Rad27p (human homolog FEN1). To obtain a genomic view of the mutational consequence of loss of *RAD27*, a *S. cerevisiae rad27*Δ strain was subcultured for 25 generations and sequenced using Illumina paired-end sequencing. Out of the 455 changes observed in 10 colonies isolated the two most common types of events were insertions or deletions (INDELs) in simple sequence repeats (SSRs) and INDELs mediated by short direct repeats. Surprisingly, we also detected a previously neglected class of 21 template-switching events. These events were presumably generated by quasi-palindrome to palindrome correction, as well as palindrome elongation. The formation of these events is best explained by folding back of the stalled nascent strand and resumption of DNA synthesis using the same nascent strand as a template. Evidence of quasi-palindrome to palindrome correction that could be generated by template switching appears also in yeast genome evolution. Out of the 455 events, 55 events appeared in multiple isolates; further analysis indicates that these loci are mutational hotspots. Since Rad27 acts on the lagging strand when the leading strand should not contain any gaps, we propose a mechanism favoring intramolecular strand switching over an intermolecular mechanism. We note that our results open new ways of understanding template switching that occurs during genome instability and evolution.

Processing Okazaki fragments during the synthesis of the lagging DNA strand is an extremely complex task. On the lagging strand, as the DNA helix is opened up, Pol α-primase first synthesizes an RNA fragment of between 7 and 14 nucleotides and elongates it with a short amount of DNA. Pol δ then extends the primer until it reaches the synthesized downstream fragment, a length of ∼200 nucleotides (Kunkel and Burgers 2008; Nick McElhinny *et al.* 2008; Burgers 2009). The polymerase then displaces the RNA primer of the previous Okazaki fragment, resulting in a 5′ single-stranded flap structure (Burgers 2009). The RNA primers must be removed from the 5′ end of the Okazaki fragments by a nuclease in order to ligate the nearby fragments into a continuous DNA molecule. Three pathways have been suggested to remove the RNA/DNA primer during Okazaki fragment maturation, and all of these pathways involve a structure-specific flap endonuclease, specifically the *Saccharomyces cerevisiae* Rad27 or its human homolog FEN1 [reviewed in Zheng and Shen (2011)]. In the first pathway, RNase H removes most of the RNA moieties, yet frequently one RNA moiety remains attached to the DNA, preventing the ligation of two adjacent Okazaki pieces. The last ribonucleotide is then removed by the 5′ exonuclease activity of FEN1 (Turchi and Bambara 1993; Turchi *et al.* 1994; Waga and Stillman 1994). However, as deletion of both yeast RNase ^1^H and RNase H2 is viable, this is probably not the primary mechanism (Frank *et al.* 1998). In the second pathway, a short 2–10 nt flap is formed following cleavage by FEN1 (Harrington and Lieber 1994; Bambara *et al.* 1997; Shen *et al.* 2005; Stith *et al.* 2008). In the third pathway, *DNA2* first shortens a long flap, and only then does FEN1 remove the flap (Bae *et al.* 1998, 2001; Bae and Seo 2000; Masuda-Sasa *et al.* 2006). After Rad27/FEN1 removes the RNA/DNA primer, a ligatable nick is formed, enabling DNA ligase 1 to immediately seal it. With successful ligation of the nick, pol δ can be recycled to continue the replication of the lagging strand.

Okazaki fragment maturation occurs as the next downstream fragment is already being synthesized, and this process needs to be in coordination with the progress of the replication fork. Even in a simple genome, such as that of *S. cerevisiae*, Okazaki fragment maturation occurs ∼100,000 times per replication cycle (and tens of million times per replication cycle in the human genome) (Burgers 2009). Due to this complexity, Okazaki fragment maturation poses a significant challenge to genome stability. However, it is still not clear if a checkpoint mechanism transduces delays in Okazaki fragment maturation and how the cell would act upon such a signal. For these reasons, mutations in the components responsible for Okazaki fragment maturation are likely to cause severe genome instability. Deletion of *RAD27* is tolerated in yeast; therefore, it is likely that an alternative but potentially less effective nuclease is capable of processing Okazaki fragments in yeast (Johnson *et al.* 1995; Reagan *et al.* 1995). Furthermore, it is not clear yet how loss of *RAD27* influences instability at the genomic level.

Based on genetic reporter assays that measure changes in the sequence of a single locus, it appears that loss of function of Rad27 in yeast leads to a unique mutation signature. First, instability of microsatellites (small tandem repeats with units 10–60 bp in length) and mini satellites (larger repeat units) has been observed, and there is a tendency toward insertions rather than deletions (Kokoska *et al.* 1998). Second, *rad27*Δ strains also accumulate insertions of up to 100 bp flanked by nontandem direct repeats (Tishkoff *et al.* 1997). Finally, *rad27*Δ strains show increased mitotic recombination (Tishkoff *et al.* 1997). Whether or not the mutational signature of *rad27*Δ is consistent across the entire yeast genome is unknown.

Here, we used whole genome sequencing of *rad27*Δ strains to detect the spectrum of mutations of short (<300 bp) events throughout the entire genome. We confirm that insertions are the most common events in *rad27*Δ strains, and events in simple repeats are as frequent as insertions mediated by direct repeats. Interestingly, *rad27*Δ strains undergo template-switching events, resulting in the creation of larger DNA palindromes. These template-switching events can be followed by DNA amplification, as well as the conversion of quasi-palindromic sequences into perfect palindromes. This latter finding was not previously reported, and to our knowledge this is one of the first examples of how whole genome sequencing of genetically modified strains can pinpoint regions that are prone to complex mutations.

## Materials and Methods

### Strains

Analyses were conducted with the *rad27*Δ haploid strain RJK56 (Kokoska *et al.* 1998), which has the MS71 background (Strand *et al.* 1995). The genotype of RJK56 is *MATα ade5-1 leu2-3 trp1-239 ura3-52 his7-2 LEU+ rad27*Δ *ρ+*. A single colony was picked from the subculture zero plate and streaked for single colonies on YPD. This plate was designated subculture one. After ∼7 d, the cells in the heavy growth were frozen away as subculture one for that colony. Another single colony was then picked and streaked for singles (subculture two). This process was then repeated for five subcultures. Cells from frozen subculture five were struck for single colonies on YPD. After 3 d, a single colony was picked from the plate and streaked for single colonies on YPD. This plate was designated subculture six. After ∼3 d, another single colony was picked and streaked for singles. This process was repeated for 25 subcultures.

### Generation and analysis of high-throughput sequencing data

Fourteen colonies were sequenced using Illumina paired-end reads: two independent colonies from subculture zero (RJK56-1-0/PG1016, RJK56-2-0/PG1017), two colonies from subculture five (RJK56-1-5/PG1018, RJK56-2-5/PG1019), and 10 colonies from subculture 25 (RJK56-1-25.[1-5]/MD1020-MD1024 and RJK56-2-25.[1-5]/MD1025-MD1029). The DNA was prepared for sequencing using the protocol recommended by Illumina for the HiSeq2000. The samples were sequenced using an Illumina HiSeq2000 machine, generating paired-end reads of 100 bp for the subculture 25 colonies and reads of 93–100 bp for the subculture zero and five colonies. For the 14 sequenced samples, coverage varied from 65- to 289-fold.

### Detecting insertions and deletions (INDELs)

INDELs were detected using a combination of two methods: Burrows-Wheeler Alignment Tool (BWA, Li and Durbin 2009) was used for reference assembly and Abyss was used for *de novo* assembly (Simpson *et al.* 2009). During BWA assembly, reads were assembled on a previously sequenced and assembled MS71 strain (Strand *et al.* 1995), which was assembled with S288c as a reference. Since MS71 shows hybrid regions that resemble both S288c and YJM789, we decided not to assemble directly on the S288c sequence. However, all positions in our analysis are presented here in SGD coordinates. SAMtools was then used to extract “pileup” files out of the assembled files (Li *et al.* 2009), creating files that show the number of bases supported at each position. Only positions that did not overlap repeats, as determined by RepeatMasker using the *S. cerevisiae* library (Jurka 2000), and positions that passed the vcf-annotate script from VCF-tools (Danecek *et al.* 2011) were considered. During Abyss assembly, since the reference assembly can identify relatively small INDELs, we applied Abyss *de novo* assembly for our data. Each of the 14 strains was analyzed with multiple *C* parameters, and the *C* value was chosen that had resulted in the combination of the contig of maximal length and of the highest N50 (the N50 is the size of the smallest of all the large contigs covering 50% of the genome). For each strain, the Abyss Scaffold file was then masked with RepeatMasker (*e.g.*, Tys and LTRs) using the *S. cerevisiae* library and Lagan.pl (Brudno *et al.* 2003) was used to place each of the *de novo* contigs into the reference chromosomes. For each strain, MAFFT (Katoh *et al.* 2009) was then used to align the contigs to chromosomes and produce an alignment. Finally, for each of the chromosomes, all strains were aligned using MAFFT with profile mode, and INDELs were extracted using in house PERL scripts. The final alignment was presented using SEAVIEW viewer (Gouy *et al.* 2010).

We looked for events that occurred in at least one of the strains that was subcultured for 25 generations but not shared among all of the subculture 25 strains. Abyss Events are only predicted if at least one of the subculture zero or five strains were assembled in the region. INDELs in telomeric regions and INDELs that overlapped Ns were also ignored. Small (<9) Abyss events were selected if they were supported by BWA (even if they did not pass the strict classification in the first list). Integrative Genomic Viewer (IGV, Robinson *et al.* 2011) was used to manually inspect all cases.

Each locus with an INDEL was assigned to the following classifications: (1) INDELs in SSRs (*e.g.*, mononucleotides, dinucleotide, *etc*.); (2) INDELs mediated by short direct repeats; (3) INDELs involved in palindromic expansion; (4) clusters of INDELs or clusters of INDELs and single nucleotide polymorphisms (SNPs) that can be explained by a quasi-palindrome correction; or (5) other INDELs. Each of these classes is further grouped to separate insertions, deletions, or unknown/mixed mutations.

Classification of the INDELs was performed using in house PERL scripts and a combination of the following programs: Repeatmasker, tandem repeat finder (TDF, Benson 1999), EMBOSS palindrome (Rice *et al.* 2000), and BLAST of a sequence against itself (Altschul *et al.* 1997). In the classifications of duplications/deletions mediated by a direct repeat, events are either of the type *ABA* to *ABABA* (insertion) or *ABABA* to *ABA* (deletion), where *A* is the direct repeat and *B* is a unique sequence (*A*, *B* are regions of DNA). The program TDF was used to identify A and B regions. The length of A+B should equal the length of the INDEL. If TDF revealed multiple choices that fit the length of the INDEL, the duplications with the highest similarity between copies was chosen. Therefore, events in nonperfect simple repeat regions can end up as insertion mediated by tandem repeat.

### Detecting mutations

Mutations were identified by two reference assembly methods: BWA and CLC Genomics. BWA mutations were identified as described above for INDELs. CLC Genomic Workbench (currently Qiagen) was also used to identify mutations. Reads from each of the 14 strains were assembled independently to the MS71 reference genome, and variant calling was performed with a minimum variant calling of 90%. A “filter against control reads” analysis was used to filter events shared with subculture five strains. IGV viewer (Robinson *et al.* 2011) was used to eliminate cases with SNPs that were shared by all strains by manually inspecting all events. SNPs in clusters with and without INDELs were tested by EMBOSS palindrome (Rice *et al.* 2000) and BLAST (Altschul *et al.* 1997) for the identification of palindromes and inverted repeats (IRs).

### Detecting template switching in Saccharomyces evolution

Inverted repeats with an arm length of at least five nucleotides and spacer up to 70 bp were identified in S288c coding genome using the EMBOSS palindrome package (Rice *et al.* 2000). An orthologous region for each IR was detected in the following *Saccharomyces* genomes: *S. eubayanus* (GCF_001298625.1), *S. uvarum* (GCA_000732305.1), *S. arboricola* (GCF_000292725.1), *S. kudriavzevii* (GCA_000167075.2), *S. mikatae* (GCA_000167055.1), and *S. paradoxus* (GCA_000166955.1). BLAST was used to identify orthologs (Altschul *et al.* 1997) requiring at least one additional gene from each side of the query genes. Multiple sequence alignments (MSAs) were reconstructed using MAFFT V3.705 (Katoh *et al.* 2009). Finally, the number of perfect but different IR variants in an orthologous region was detected and compared to the number of different perfect IRs in 1000 simulated MSAs. Note, the evolutionary simulation was performed without a template-switching mechanism.

### Data availability

Strains are available upon request. A list of all events reported is provided in Supplemental Material, File S1. The sequencing data have been deposited in the National Center for Biotechnology Information Sequence Read Archive (accession no. SRP117788).

## Results

**Figure 1:**
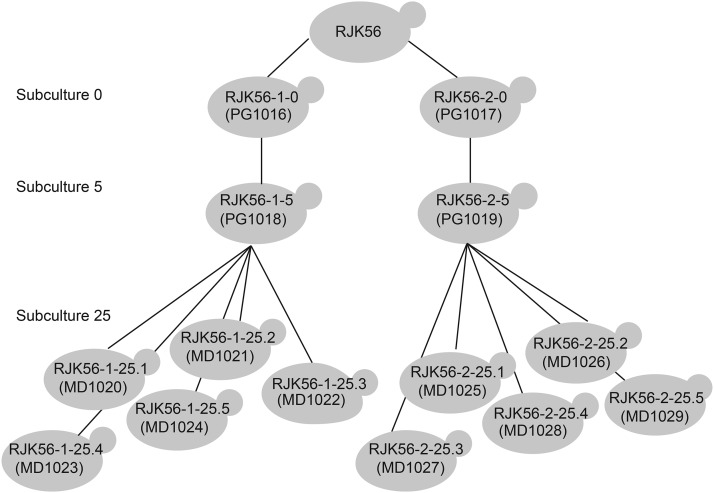
The experimental design of this study. The initiator strain was the RJK56 *rad27*Δ haploid (Kokoska *et al.* 1998). Two subculture zero plates were used and streaked for single colonies on YPD and grown for ∼7 d. One colony from each of these plates was designated subculture one, and, after growing and streaking for singles, one colony from each plate was picked to be grown as subculture two. We repeated this process until subculture five. After subculture five was plated for single colonies, five independent colonies were picked, and the process was repeated until subculture 25, with each subculture grown for ∼3 d. Using a HiSeq2000 machine, we sequenced two lines, each with seven colonies, for a total of 14 strains. Each line contained one sample from subculture zero, one sample from subculture five, and five samples from subculture 25.

Mutations in *RAD27* and other strains are typically studied using a single gene for which forward mutations can be selected. The rate of changes in this one gene, as well as the spectrum of mutations, is assumed to be consistent across the entire genome. Here, we used whole genome sequencing of *rad27∆* strains to directly test the mutation spectrum. A single *rad27∆* haploid colony (RJK56, Kokoska *et al.* 1998) was picked from a subculture zero plate streaked for single colonies on YPD that was grown for ∼7 d. A colony from this plate was designated subculture one. Restreaking and growth on YPD was repeated until subculture five was obtained. Five independent colonies were picked from the subculture five plate, and each of these colonies was further streaked until subculture 25, growing each subculture for ∼3 d. Using Illumina deep sequencing technology, we sequenced two separate lines, each with the following seven colonies: one from subculture zero, one from subculture five, and five from subculture 25, for a total of 14 strains (Figure 1). HiSeq2000 was used to analyze the data. The genomic analysis focused on the spectrum of events ranging in size from 1 to 300 bp.

We identified 455 loci with changes that occurred in at least one of the strains that was subcultured for 25 generations but were not shared among all of the subculture 25 strains (File S1). The events were classified to different mutation categories as described below. The rate of mutation for each category was calculated as rate per colony/generation/base pair. The information is provided in File S1. Importantly, we provide a minimal estimation for the rates because some of the events can be reversal and some of them that present in multiple strains could have been created independently. The majority of events we observed were INDELs. Two types of INDELs were most common: INDELs in simple sequence repeats (SSRs, 183 loci) and INDELs mediated by short direct repeats (177 loci). An additional class consisted of INDELs and SNPs that are presumably the result of template-switching events, a class that was not previously associated with *rad27∆* strains (21 events). Other events were composed of SNPs and gene conversions between paralogous genes.

**Figure 2:**
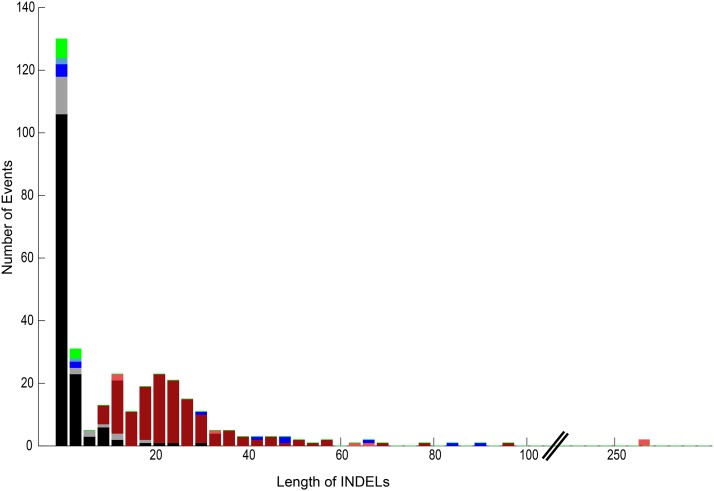
Stacked histogram depicting the lengths of 338 INDELs that appear only once in the 10 *rad27*Δ subculture-25 strains. The different classes of events include insertions (black) and deletions (gray) in SSRs, insertions (red) and deletions (orange) mediated by direct repeats, insertions (blue) and deletions in template-switching events, and other insertions (green).

Among INDELs, insertions are more common than deletions (Figure 2). Most events were unique, *i.e.*, they appeared in only one strain; however, 51 of the INDELs and 4 of the template-switching loci were found with events in multiple strains, indicating that these loci are prone to changes. Here we focus on INDELS mediated by short directed repeats (Tishkoff *et al.* 1997) and template switching. The full list of all events depicted are available in File S1.

### rad27∆ strains have hotspots for changes

**Figure 3:**
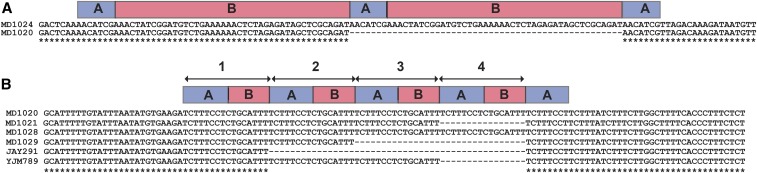
(A) An example for an insertion mediated by a direct repeat. The top sequence in this pairwise alignment includes an insertion of 51 bp compared to the other strains (represented here by a single strain MD1020). The bottom sequence represents the ancestral form with the structure of *ABA*, and the top sequence with the insertion has the structure of *ABABA* (SGD position 470,500 on chrXIII). (B) Three types of changes at ChrII:392888 that were observed in *rad27*Δ strains. A unit of 16 bp composed of an 8-bp direct repeat (indicated as *A*) and an 8-bp unique sequence (indicated as *B*). Most strains, as well as the two zero and two five-strains, included the ancestral form of three copies of *AB* (represented here by strain MD1021). However, two independent strains (MD1020, MD1028) included four copies of *AB*, and one strain (MD1029) included two copies of *AB*. *S. cerevisiae* strain JAY291 has one *AB* unit, and YJM789 contained three *AB* units in this locus.

In the case of insertions mediated by a direct repeat, the nonmutated, original locus is the form *ABA*, where *B* is a unique stretch of DNA sequence flanked by two short direct repeats, marked as *A* (Figure 3A, bottom sequence). After the insertion has occurred, the mutated locus would be in the form of *ABABA* (Figure 3A, top sequence). In the case of deletions, the opposite scenario occurs; *ABABA* is transformed to *ABA*. For insertion events, the size of the unique region *B* can range in size between 1 and 91 bp, and the size of the repeat *A* can range between 1 and 21 bp. This is a somewhat larger size range compared to the previously detected (Tishkoff *et al.* 1997) 5–108 bp, which was mediated by a repeat of 3–12 bp.

One interesting example occurs around position 392,888 on chrII, between the *SML4* and *ECM33* genes (Figure 3B). This locus includes three variants with two to four copies of a 16-bp AB unit. This example, verified by PCR, contains duplications of a 16-bp unit mediated by a 8-bp direct repeat (A) in two independent strains (RJK56-1-25.1/MD1020, RJK56-2-25.4/MD1028) and a deletion of 16 bp mediated by a direct repeat in an additional strain (RJK56-2-25.5/MD1029). Moreover, BLAST analysis of additional *S. cerevisiae* strains indicated that variation in this locus is common and that different wild-type strains have variation in the number of repeats. For example, one single AB copy is observed in the JAY291 strain that is used in bioethanol production, and three AB copies are observed in the YJM789 strain that was derived from the lung of an AIDS patient (Wei *et al.* 2007).

**Figure 4:**
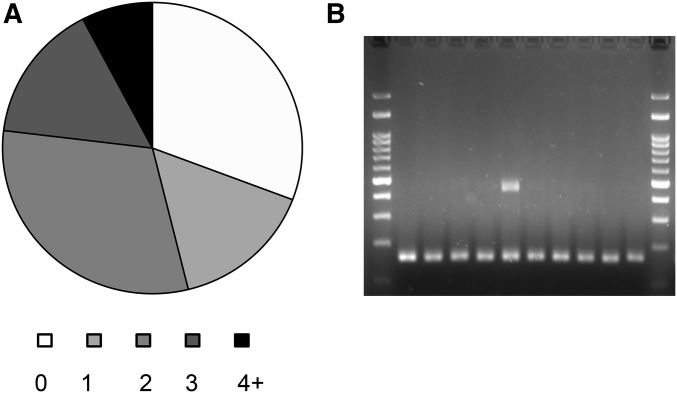
Structural variation hotspots in *rad27*Δ strains. (A) Several loci were shown to be different from the consensus sequence in multiple strains. A pie chart showing the number of variants differ from the consensus strain among 10 randomly picked colonies that were subcultured from two zero- and two five-strains in these loci is presented. In 70% of the 13 cases, at least one of the picked colonies showed the alternative length of a PCR product. (B) An example of an alternative length PCR product of one of the isolates from subculture zero at the locus 72410 on Chromosome II.

The appearance of identical events in the two independent *S*. *cerevisiae* lines is striking. The majority of events that appear in multiple strains are insertions mediated by direct repeats (29); 17 of the events are in SSR and 4 are template-switching events (see below). We analyzed 16 events that appear in multiple strains of which 13 were PCR-validated to appear in multi strains; one was identified to appear only in one strain, while another two events we analyzed were not resolved. Since events repeated in several isolates we hypothesized that the sites are prone to changes. To test this hypothesis, we streaked for singles colonies from subcultures zero and five from both lines and randomly picked 10 colonies from each of the four, a total of 40 colonies. In 70% of the 13 cases, at least one of the picked colonies showed an alternative length of the PCR product. As can be seen in Figure 4B, we could find evidence for sectoring: both the original allele and an evolved one appear in a culture grown from a single colony isolate. Thus, these events are highly unstable and during subculturing they can appear and disappear (Figure 4). To the best of our knowledge, this is one of the first examples of how NGS data provide prediction to loci of structural variability in the population.

### rad27∆ strains show template-switching events

A template-switching mechanism where the replication fork presumably hops between templates on two DNA strands results in the most interesting class of events. Template-switching mediated events were found at a total of 21 loci (File S1), of which 17 events were unique, and four events were detected in multiple strains. We verified by Sanger sequencing or PCR 15/16 unique template-switching events. The four template-switching events appearing in multiple strains were also verified as noted before. These events resulted in the following outcomes: (a) a cluster of SNPs and a combination of INDELs and SNPs that was formed by template switching between either the arms of imperfect IRs or quasi-palindromes, leading to the creation of a perfect IR or palindrome (10 events); (b) long insertions that were created by a combination of palindrome extensions and DNA amplification (six unique events, four events in multiple strains); and (c) palindrome expansion followed by exonuclease activity without DNA amplification (one event).

**Figure 5:**
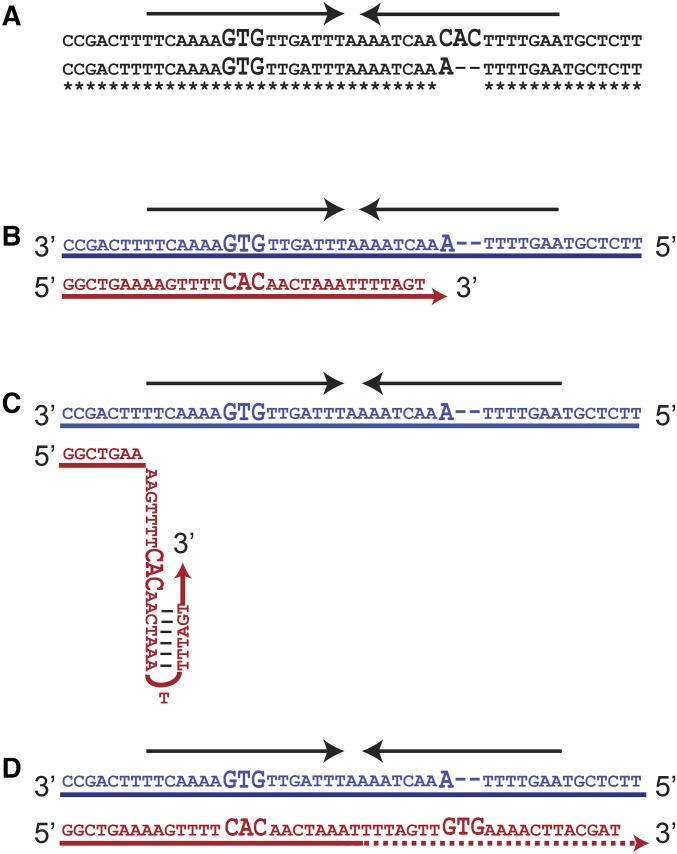
Quasi-palindrome to palindrome correction in *rad27*Δ strains. (A) Variation between two *rad27*Δ strains from subculture 25. The top sequence represents the mutated strain that includes a perfect palindrome, and the bottom sequence represents the original (nonmutated) strain that includes a quasi-palindrome. (B) Replication starts on a template similar to the bottom strain in A. The fork synthesizes one arm and starts synthesis on the second arm (C). Then, the newly synthesized strand folds back using the homology between the arms. A template switch occurs, causing the nascent strand to use itself as a template, eliminating variation between the arms of the palindrome. Finally, (D) the nascent strand returns to the correct template. The next round of replication results in two daughter cells that resemble the variants depicted in A. Arrows indicate palindromes, and bases showing the differences between arms are shown in large font. The red strand is the nascent strand and the blue strand is the original template strand.

Quasi-palindrome is a palindromic or IR sequence that bears mutations in one of the arms. A simple example is *5′AGAACAxxxxTcTTCT 3′* where *c* shows a mismatch between arms and *x* represents a spacer base. A conversion of this quasi-palindrome to a perfect palindrome means an appearance of identical arms in inverted orientations, thus 5′ AGAACAxxxxTGTTCT 3′. A mechanism of template switching resulting in a quasi-palindrome to perfect palindrome correction is illustrated in Figure 5. Two subculture 25 *rad27∆* strains are shown in Figure 5A. The top sequence is mutated and has a perfect IR with identical arms of 16 bp separated by a spacer of 1 bp. The bottom strain, which represents the original sequence, has an imperfect IR. The arms of the imperfect palindrome are different from each other in three points (one mismatch and a deletion of two bases, all of which are shown in large font in Figure 5). The most parsimonious way to explain how the top sequence originated is that the replication fork stalled and switched templates; the stalled replication fork folded back used the nascent DNA strand as a template. As shown in Figure 5B, replication starts using a template with the sequence of the bottom strain in Figure 5A. The newly synthesized strand replicates the template of the 3′ arm of the IR and then begins replicating the second arm but stalls. Next, the newly synthesized strand forms a secondary structure, resulting in a template switch, where the recently synthesized IR arm is used as the template (Figure 5C). Finally, the replication fork realigns to the legitimate template (Figure 5D). At the end of this process, the bottom strand in Figure 5D would resemble the top and mutated strain in Figure 5A, but the top strand on Figure 5D would resemble the bottom and original strain in Figure 5A. At the next round of replication, two different daughter cells will be produced, one with and one without a perfect IR (as shown in Figure 5A).

**Figure 6:**
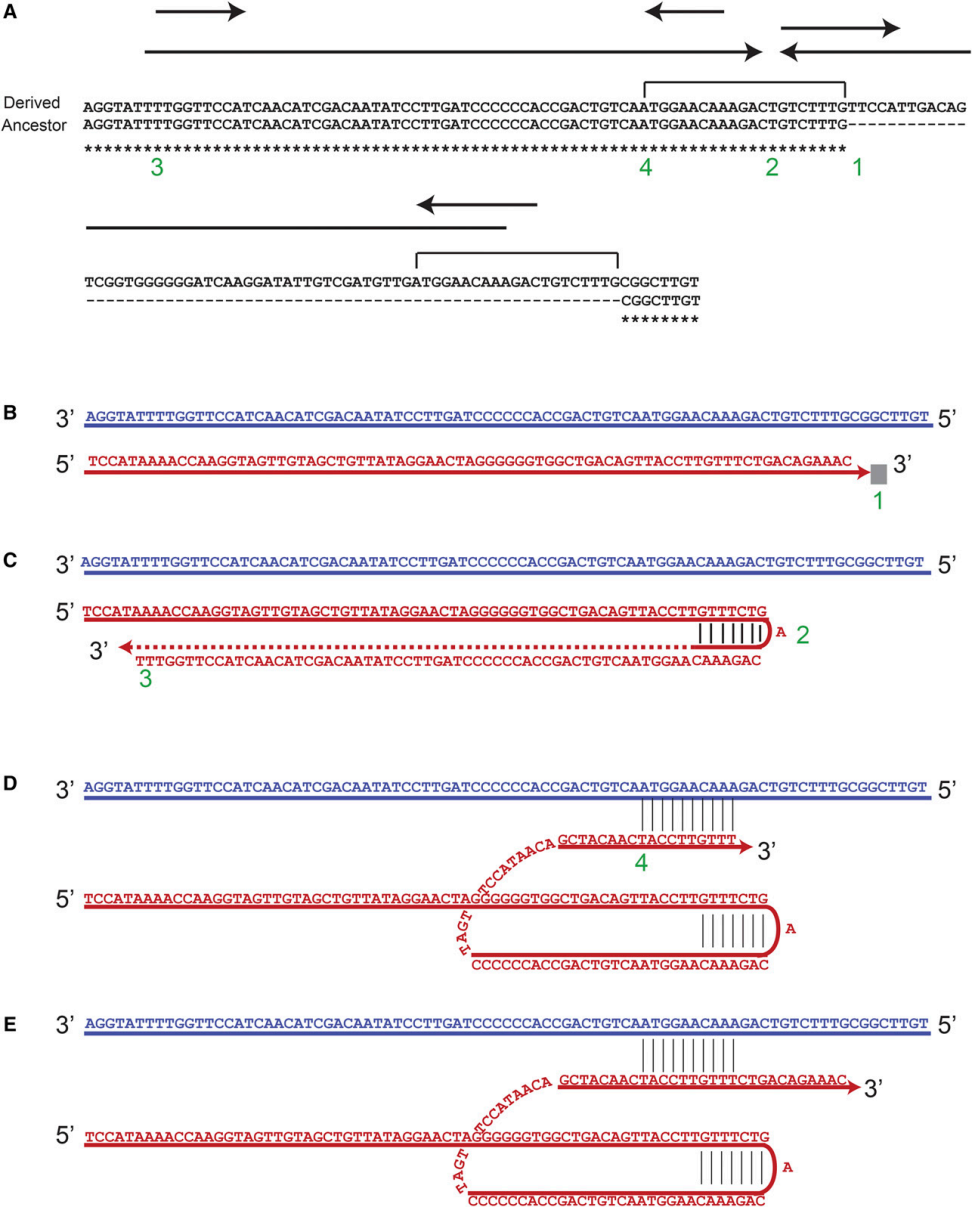
Palindrome expansion occurs via template switching in *rad27*Δ strains. Insertion at ChrII 715496. (A) The top line includes the mutated strain with an insertion mediated by a palindrome; the bottom line is the original sequence. Arrows indicate palindromes, and brackets indicate duplications. Numbers indicate important points in the mechanism. (B) DNA synthesis starts on a template similar to the bottom line in A and continues until the fork is stalled (point 1). (C) At point 2, the DNA folds back (template switch #1) and synthesizes until it arrives at point 3. (D) The synthesized strand aligns back to the chromosome at point 4 (template switch #2) and (E) continues synthesis in the original direction. The top strand resembles the bottom and original strain in A, and the bottom strand resembles the top and mutated strain in A. The next round of replication results in two daughter cells with the rearrangements depicted in A. The red strand is the nascent strand and the blue strand is the original template strand.

**Figure 7:**
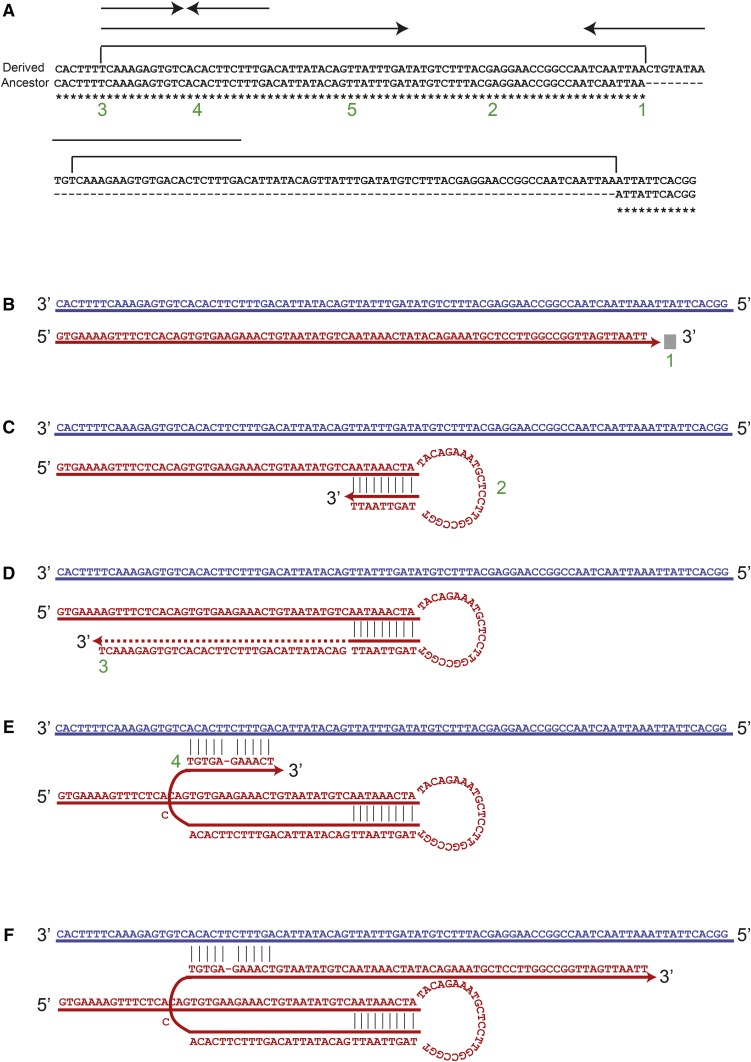
Palindrome-mediated DNA amplification occurs via template switching in *rad27*Δ strains. In this example, found at chrVII: 657,551, DNA amplification is the dominant outcome. (A) Two different subculture 25 strains are shown: the top line is the mutated sequence and includes an insertion mediated by a palindrome, and the bottom line is the original sequence. Arrows indicate palindromes, brackets indicate duplication, and numbers indicate important points in the mechanism. (B) DNA synthesis starts on a template similar to the bottom line in A and continues until the fork is stalled (point 1). (C) At point 2, the DNA folds back (template switch #1) (D) and synthesizes until it arrives at point 3. (E) The nascent strand aligns back to the top strand (template switch #2) at point 4 (F) and continues synthesis in the original direction. The top strand resembles the bottom and original sequence in A, and the bottom strand resembles the top and mutated sequence in A. The next round of replication results in two daughter cells with the rearrangements depicted in A. The red strand is the nascent strand and the blue strand is the original template strand.

We also found template-switching events that caused DNA amplification and extension of the palindrome. The ratio of the length between the palindrome or IR expansion and the amount of DNA amplification varied between the events, depending on the point where the replicated strand returned to the original template. In some examples, like in Figure 6, the palindrome expansion segment is bigger than the amount of DNA amplification, while in other events, like Figure 7, the opposite relationship was observed.

In Figure 6A, two variants of subculture 25 *rad27∆* strains are depicted; the top strain contained the mutated sequence, which includes an insertion of 66 bp, the result of palindrome expansion. The bottom strain, which represents the original sequence, includes a palindrome of 15 bp with a center at point 2. The mechanism of mutation generation here is best explained by a folding-back mechanism that involves two steps of template switching. First, while replicating on a template with the sequence of the bottom strain, the replication fork is presumably blocked at point 1 (Figure 6B). The nascent strand is then folded back to the center of a small, 15-bp palindrome (point 2, Figure 6C) and synthesis occurs until point 3. After replicating a total of 55 bp, the nascent strand realigns back to the original template (Figure 6D) and replication resumes using the original template (Figure 6E). This transaction results in a 66-bp insertion and a short duplication. At the end of this process, the bottom strand in Figure 6E would resemble the top and mutated strain in Figure 6A, while the top strand on Figure 6E would resemble the bottom and original strain on Figure 6A. The next round of replication will result in two daughter cells with structures similar to the two forms in Figure 6A.

A similar event was found at the *NNF2* gene, near ARS727, and is shown in Figure 7. Here, a duplication makes up a larger part of the insertion, although palindrome expansion is also involved. The top sequence in Figure 7A represents that of a mutated strain from subculture 25 with an insertion, and the bottom sequence in Figure 7A represents the original form prior to insertion and was found in the other subculture 25 strains. First, while replicating on a template similar to the bottom strain in Figure 7A, the replication fork stalls (Figure 7B, point 1 in Figure 7A). Next, the nascent strand folds back (Figure 7C, point 2 in Figure 7A) forming a stem loop structure due to imperfect IRs that are centered at point 2. DNA synthesis continues up to point 3 (Figure 7D). The fork realigns to the original template at point 4 (Figure 7E) and replication resumes (Figure 7F). At the end of the process, the bottom strand will resemble the top and mutated strain on Figure 7A, while the top strand on Figure 7F will resemble the bottom and original strain in Figure 7A. The next round of replication will result in a daughter cell with the insertion. We note that it is also possible that the fork does not return to the native template at point 4 but may fold back on itself a second time, using the short IRs (indicated in Figure 7A). In this case, the fork would realign to the original template strand at point 5. Both options lead to the same final sequence.

**Figure 8:**
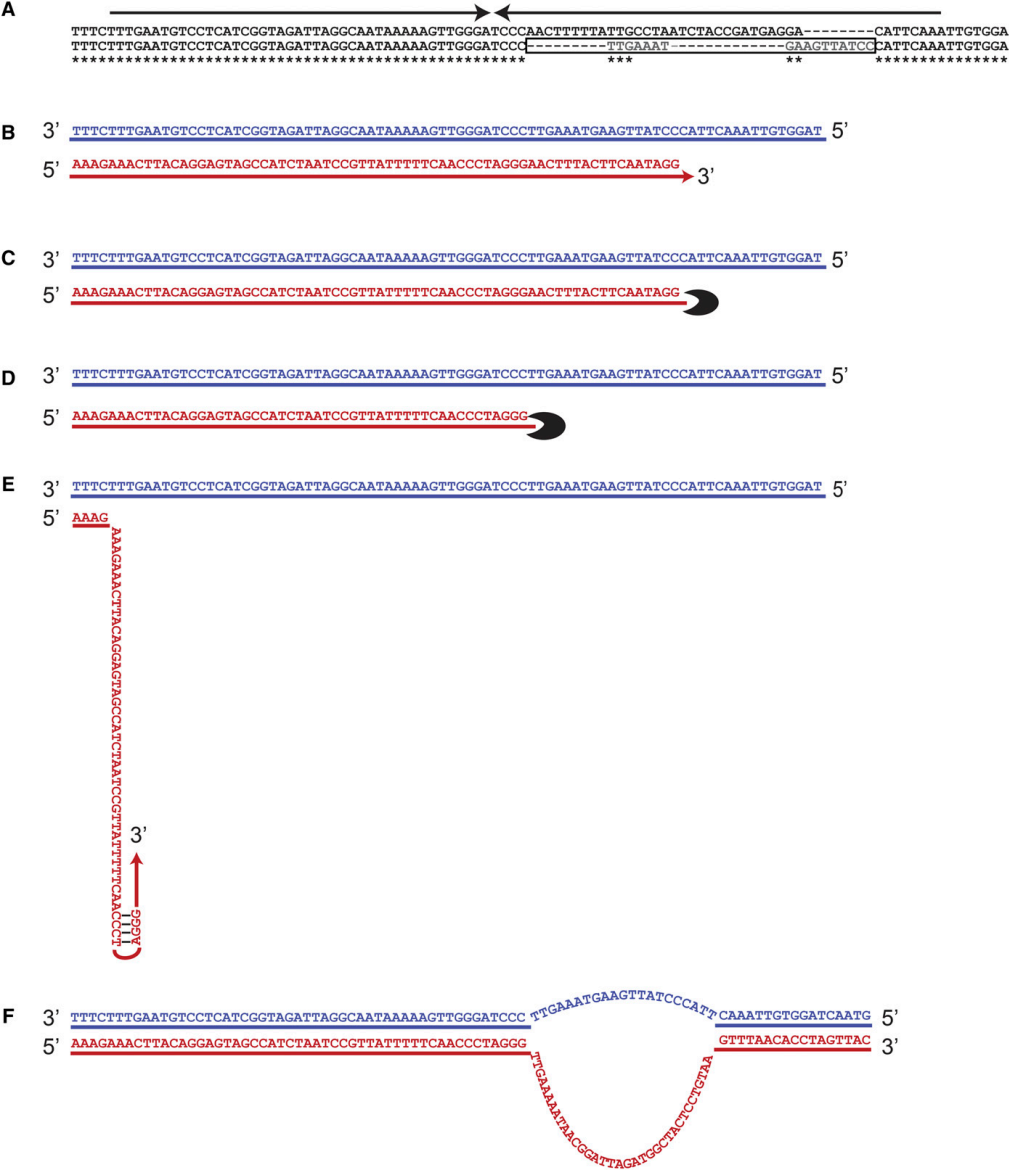
Nascent strand processing allows palindrome amplification via template switching in *rad27*Δ strains. (A) Two different subculture 25 strains are depicted, and the top sequence is mutated and includes a palindrome expansion. The bottom sequence is unchanged and includes an additional 17 bp (boxed). Arrows indicate palindromes. (B) DNA synthesis starts on a template similar to bottom sequence in A and continues until the fork is stalled. (C and D) An exonuclease then removes 17 bases. (E) The newly synthesized strand folds due to the homology between arms. A template switching occurs causing the nascent strand to use the recently synthesized arm as a template, expanding the palindrome. (F) The two DNA strands now differ in the area of expansion: the top strand resembles the bottom and original sequence in A, and the bottom strand resembles the top and mutated sequence in A. The next round of replication results in two daughter cells with the sequence changes that appear in A. The red strand is the nascent strand and the blue strand is the original template strand.

A fourth example of template switching without DNA amplification is shown in Figure 8. Two subculture 25 strains are shown in Figure 8A. The top strain represents the mutated strain including a palindromic insertion, and the bottom strain in Figure 8A represents the original sequence. Here, the original sequence includes 17 bases that are missing in the mutant strain. The most parsimonious explanation is that these 17 bases were deleted by an exonuclease during the process of palindrome expansion. Removal of these bases enables the newly synthesized strand to fold back and form a secondary structure thus switching template and replicating the same strand resulting in palindromic expansion. The process leads to a bottom strand similar to the mutated strain and a top strand similar to the original strain. The next round of replication will result in two daughter cells with these different genotypes.

### Template-switching events through Saccharomyces evolution

**Figure 9:**
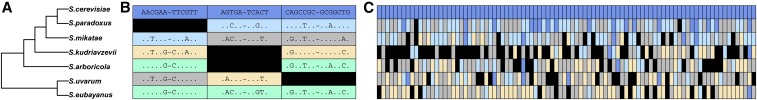
Evidence for quasi-palindrome to palindrome conversion via template switching during evolution of *S. cerevisiae*. (A) A phylogenetic tree of seven *Saccharomyces* genomes. (B) Three perfect palindromes appear in S288c three coding regions. Different perfect palindromes appear at the same loci in three additional genomes. Each locus is shown in a column and different colors show different palindrome forms. Loci that are in a quasi-palindromic state are shown in gray. Cases in which the loci could not be identified are shown in black. Positions identical to S288c are shown with a dot and the spacer between arms is represented with a tilde sign. (C) Ninety-two S288c loci that appear in two additional genomes.

We found 21 events of template switching in *rad27∆* isolates. Such events were not reported for WT yeasts (Lynch *et al.* 2008; Nishant *et al.* 2010). Is template switching a phenomenon affecting normal yeast evolution? To answer this question, we looked for evidence of template switching causing quasi-palindrome to palindrome correction resulting in different but perfect orthologous IRs through *Saccharomyces* genes. We searched the SGD S288c reference genome for perfect IRs with arms of at least 5 bp and a spacer of a maximum 70 bp. For each perfect IR in S228c genes, we looked for the appearance of a slightly different perfect repeats in orthologous loci in seven fully sequenced *Saccharomyces* genomes with known phylogeny (Figure 9A). Out of 49,912 perfect palindromes in 543 genes, 3741 in 502 genes appear with at least two forms through phylogeny. This number includes 3 loci that appear in 4 forms through phylogeny (Figure 9B), and 92 that appear in 3 forms through phylogeny (Figure 9C). When comparing to an evolutionary simulation assuming no template-switching mechanism, 431 out of the 502 were statistically significant (*p* value <0.05). The meaning of such findings is that independent events of quasi-palindrome to palindrome conversion had occurred multiple times in the same locus during evolution. Thus, template switching is not only significant in the case of instability conditions, as in the case of *rad27∆*, but also as a source of variation throughout normal yeast evolution.

## Discussion

### rad27∆ strains have hotspots for changes

**Figure 10:**
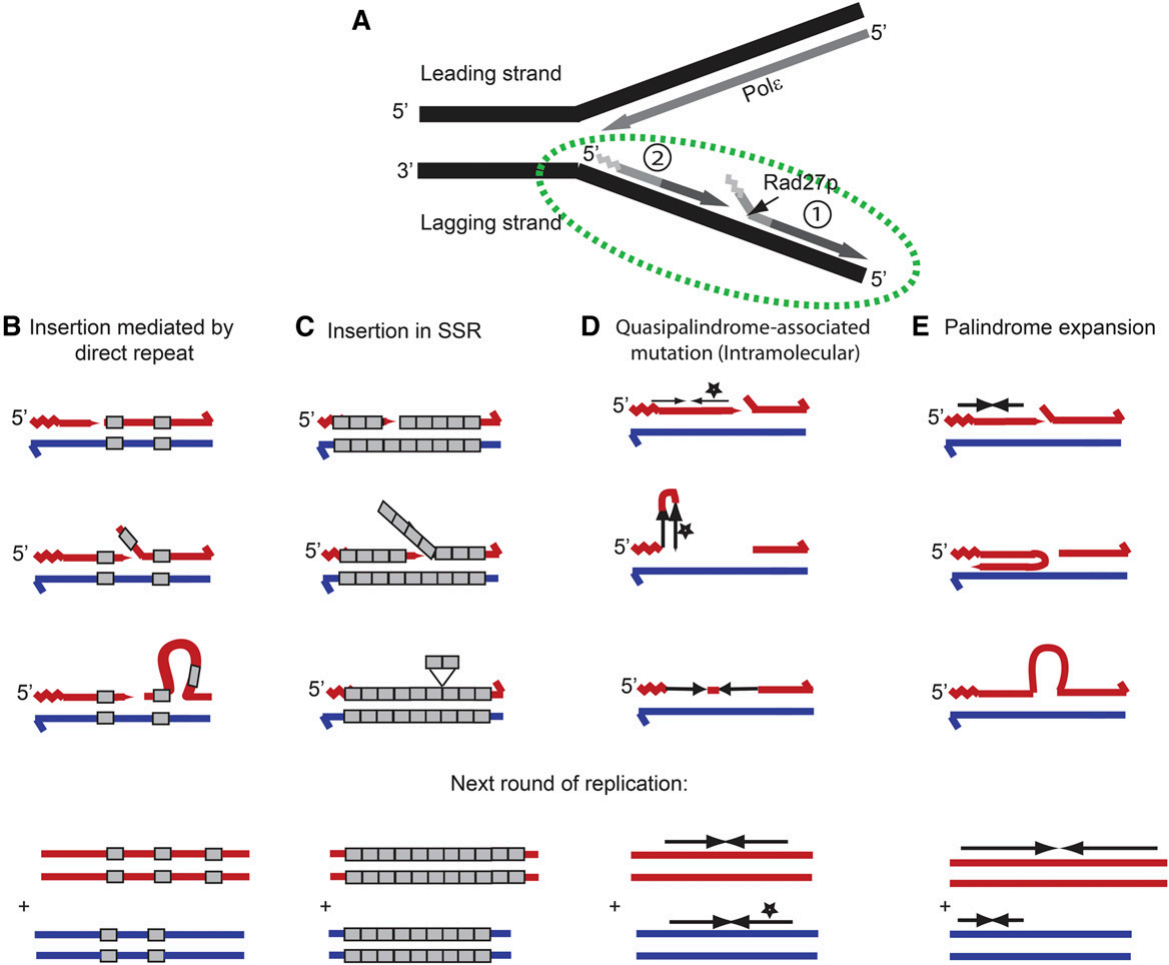
Models for the formation of the common events observed in *rad27*Δ strains. (A) Description of Okazaki fragment maturation. Primers produced by polymerase α are shown in a light gray zigzag, and the extension by polymerase δ is represented by a darker shade of gray. The predicted position where Rad27/FEN1 acts to remove the RNA/DNA primer in wild-type strains is indicated by the small black arrow. The junction between two adjacent Okazaki fragments is enlarged in B–E (green ellipse). (B) A model for an insertion mediated by a direct repeat, where realignment of the direct repeat in the wrong location enables the formation of a loop containing the unique sequence that forms the insertion in the next round of replication. (C) A model for an insertion of one repeat in SSR. We note that mechanisms B and C are similar, as the repeat can easily realign in multiple places. (D) An intramolecular template-switching model for the correction of a quasi-palindrome to a palindrome. The star indicates a change between the two copies of the IR. (E) A model for expansion of a palindrome through a template-switching mechanism. We suggest that the polymerase is stalled due to stabilization of the flap as a hairpin in the previously synthesized Okazaki fragment, or due to inefficient strand displacement. The polymerase continues synthesis using the nascent strand as a template. The red strand is the nascent strand and the blue strand is the original template strand.

Rad27 is a key player in the assembly of Okazaki fragments on the lagging strand (Figure 10A). Insertions mediated by tandem repeats are common in *rad27∆* strains (Figure 10B) (Tishkoff *et al.* 1997). We found that there are genomic hotspots for these insertions in this genotype. We note that a model of an insertion of one repeat unit in SSR is similar (Figure 10C), as the repeat can easily realign in multiple places. Figure 10B shows a model for an insertion mediated by a direct repeat, where realignment of the direct repeat in the wrong location enables the formation of a loop containing the unique sequence that forms the insertion in the next round of replication.

### Stalled replication forks in rad27∆ strains are resolved by template switching

The current view of Okazaki fragment maturation assumes a competition between DNA polymerase and DNA ligase I on the 3′ OH of the nascent strand. In the absence of Rad27, the formation of ligatable nicks is severely reduced and thus the polymerase can rebind the 3′ OH and continue synthesis (Burgers and Kunkel 2017). Our results indicate that when the legitimate single-stranded DNA template is shrinking, the polymerase may switch to other available templates rather than continue and displace the downstream Okazaki fragment. This is in agreement with biochemical data suggesting that strand displacement by pol δ is minimal (Burgers and Kunkel 2017). Therefore, here we relate to the point where the template of DNA synthesis is no longer the original template as a point of stalled replication fork. It is possible that *in vivo* strand displacement can progress further than the point of template switching but the nascent DNA is processed by nuclease as described here in Figure 8 and by Jin *et al.* (2001).

The most powerful aspect of using whole genome sequencing in the study of mutagenesis is to reveal the contribution of the sequence context. Detecting that *rad27∆* strains undergo template switching was only possible because of the use of whole genome sequencing. Moreover, a previous analysis of *rad27* null mutants was unable to detect these events (Serero *et al.* 2014). Quasi-palindrome to palindrome correction in one arm of an IR, which then serves as a template for the synthesis of the second arm, has been demonstrated in multiple organisms including bacteria and yeasts (Bzymek and Lovett 2001; Lisnić *et al.* 2005). This phenomenon has been linked to both mutational hotspots (Rosche *et al.* 1998; Viswanathan *et al.* 2000) and genetic diseases (Bissler 1998). It was also shown that obstacles in Okazaki fragments, such as RNA residues in the DNA of DNA-RNA hybrids, can also lead to template-switching events (Kim *et al.* 2013).

Either an intermolecular or an intramolecular template-switching model could be responsible for quasi-palindrome to palindrome correction (Ripley 1982; Lovett 2004). In the intermolecular strand switching model, the unpaired 3′ end of the nascent strand pairs with a single-stranded template on the opposite strand. As Rad27p is assigned to the lagging strand and acts when the leading strand should not contain any gaps, a mechanism of intermolecular strand switching is very unlikely, and we prefer a mechanism of intramolecular strand switching (Figure 10D).

A template-switching mechanism similar to that of a quasi-palindrome correction can also achieve palindrome expansion. In the absence of *RAD27*, the legitimate DNA template can be blocked. Recent data strongly argue that the replication fork is dynamic and can adopt alternative templates when it is blocked to allow completion of DNA synthesis, even at the risk of a severe genome rearrangement (Lee *et al.* 2007; Weinert *et al.* 2009). Here, at least two alternative templates can be proposed for strand misalignment at the border between the newly synthesized Okazaki fragment and the previous fragment. First, when *RAD27* is missing, the flap structure of the old Okazaki fragment can create a hairpin, preventing the strand displacement by pol δ (Figure 10E). Therefore, the newly synthesized Okazaki fragment will fold back and synthesize in the opposite direction. It was shown that palindromes can also be achieved when a double strand break occurs next to a short inverted repeat (Butler *et al.* 1996; Rattray *et al.* 2005) or by origin-dependent IR amplification (Brewer *et al.* 2011). Forming longer palindromes can also be unstable (Butler *et al.* 1996; Tanaka *et al.* 2002; Brewer *et al.* 2011), and their formation is associated with gene amplification events that can promote or contribute to carcinogenesis (Narayanan *et al.* 2006; Tanaka *et al.* 2007; Tanaka and Yao 2009).

In breast cancer, it was shown that palindrome formation is an early stage in gene amplification (Tanaka *et al.* 2005; Tanaka and Yao 2009), often associated with poor prognosis (Guenthoer *et al.* 2012). Similarly, in our *rad27*Δ analysis we found that palindrome expansion is primarily associated with DNA amplification. We note, however, that gene amplification in cancer includes also events much longer than we can detect in our *rad27*Δ system. In a broader context, our data support a more frequent phenomenon of primer extension when the template is unavailable, presumably due to a stalled replication fork, that causes the polymerase to switch to the nascent strand and use it as the template (Lee *et al.* 2007; Weinert *et al.* 2009).

We also show the first evidences of perfect IR representation in orthologous regions through *Saccharomyces* evolution. This finding suggests that a template-switching mechanism is important not only in instability conditions but also through evolution and normal organism life.

Finally, our results provide a mechanistic insight. It has not been clear how the 3′ end of an Okazaki fragment transfers between the polymerase and DNA ligase. The fact that we observe significant template switching indicates that the absence of a natural DNA template does not serve as a signal for the polymerase to fall off and cease DNA synthesis. It is tempting to speculate that Rad27p itself may have a direct role in signaling for the switch between the polymerase and the ligase.

## Supplementary Material

Supplemental material is available online at www.g3journal.org/lookup/suppl/doi:10.1534/g3.117.300262/-/DC1.
